# Interaction of Serum-Derived and Internalized C3 With DNA in Human B Cells—A Potential Involvement in Regulation of Gene Transcription

**DOI:** 10.3389/fimmu.2019.00493

**Published:** 2019-03-19

**Authors:** Mariann Kremlitzka, Alicja A. Nowacka, Frida C. Mohlin, Pradeep Bompada, Yang De Marinis, Anna M. Blom

**Affiliations:** ^1^Division of Medical Protein Chemistry, Department of Translational Medicine, Lund University, Malmö, Sweden; ^2^Genomics, Diabetes and Endocrinology, Department of Clinical Sciences Malmö, Lund University, Malmö, Sweden

**Keywords:** complement C3, internalization, DNA, histones, gene transcription

## Abstract

Beside its classical role as a serum effector system of innate immunity, evidence is accumulating that complement has an intracellular repertoire of components that provides not only immune defense, but also functions to maintain cellular homeostasis. While complement proteins, mainly the central component C3, have been detected in B cells, their exact function and source remain largely unexplored. In this study, we investigated the expression and origin of intracellular C3 in human B cells together with its role in B cell homeostasis. Our data provide evidence that endogenous expression of C3 is very low in human B cells and, in accordance with the recent publication, the main origin of intracellular C3 is the serum. Interestingly, we found that both serum-derived and purified C3 are able to enter the nucleus of viable B cells, suggesting its potential involvement in regulation of gene transcription. ELISA, gel shift assay, confocal microscopy, and chromatin immunoprecipitation proved that C3 and C3a strongly bind to nuclear DNA, and among the interacting genes there are key factors of lymphocyte development and differentiation. The strong interaction of C3 with histone proteins and its potential ability to induce chromatin rearrangement suggest that C3/C3a might regulate DNA transcription via chromatin remodeling. Our data reveal a novel, hitherto undescribed role of C3 in immune cell homeostasis, which further extends the repertoire how complement links innate and adaptive immunity and regulates basic processes of the cells.

## Introduction

The complement system has originally been viewed as a serum effector system of innate immunity, with components synthesized mainly in the liver and functioning to protect against invading pathogens. For a long time, complement activation was considered to take place almost exclusively in the blood, resulting in cleavage of the central complement component C3 into C3a and C3b. While the smaller fragment, C3a induces inflammation, C3b allows further propagation of the cascade leading to either lysis of the target cells or via opsonization, clearance of self, and non-self structures (1–3). Besides being an indispensable part of innate immunity, complement also functions as a link between innate and adaptive immunity (4, 5). According to the classical view, complement regulates adaptive immunity via opsonization, leading to enhanced uptake and presentation of antigens by dendritic cells with subsequent activation of T and B lymphocytes (6). The role of complement in driving adaptive immune responses is supported by the abnormal T and memory B cell response, low antibody titers and severe pyogenic infections of C3-deficient patients and mice (6–9).

Beside the traditional view, evidence is accumulating that complement has also an intracellular repertoire of components that provide not only immune defense, but also functions to maintain cellular homeostasis and mediate adaptive immune responses (10, 11). The role of intracellular “complosome” in regulation of T cell immunity has been extensively studied in the recent years. Resting T cells contain intracellular stores of C3, which is cleaved into C3a and C3b by cathepsin L (12, 13). While the smaller activation product, C3a mediates homeostatic survival signal, binding of autocrine C3b to surface CD46 results in up-regulation of cell metabolism and NLRP3 inflammasome activation, two key processes, which are indispensable for both initiation and contraction of Th1 immune responses (14–16). The C3-mediated metabolic reprogramming is essential to tip the balance between effector and regulatory T cell pools and hence, provides a novel entry point for therapies of T-cell mediated autoimmune diseases (12). Additionally, involvement of intracellular C3 in apoptotic cell handling and autophagy have been demonstrated recently, highlighting that the intracellular complosome may have a broad physiologic significance in regulation of inflammation and immune homeostasis (17–20).

The role of extracellular C3 cleavage products in driving B cell immunity is essential for both antibody secretion and development of immunological memory (6, 8, 21). The indispensable role of extracellular C3 in B cell immunity is supported by low antibody level, lack of memory B cells and severe pyogenic infections observed in C3-deficient patients (6, 22). Beside the role of extracellular C3, recent data indicate that B cells also contain intracellular stores of C3, however, its exact function and source remained largely unexplored. The first observation of C3 in B cells reported detection of C3 and C3a by confocal imaging in a wide variety of immune cells, among them blood-derived B lymphocytes (12). These results were later confirmed by Elvington et al. showing presence of C3 in freshly isolated blood-derived B cells (23). Interestingly, the protein was absent in the malignant B cell lines. Since one of the differences between freshly isolated cells and malignant cell lines is the time of culture in absence of human serum, the main source of C3, the authors concluded that the main origin of C3 in B lymphocytes—and in other cells—is the blood (23). These results indicate that intracellular C3 may have two sources: endogenous C3, which is expressed by the cells and exogenous C3, which is taken up from the surrounding milieu. Endocytosed C3 may undergo cleavage into C3a and C3b, complementing thereby the intracellular C3 pool, highlighting the continuous interaction between the intra- and extracellular complement systems.

Although intracellular C3 has been evidenced in B cells, one should consider that the B cell receptor itself can activate complement, resulting in covalent attachment of C3 fragments to complement receptors and possibly also to other acceptor sites on the cell surface (24–26). The membrane-bound and cytoplasmic C3 however cannot be distinguished via conventional Western blot methods performed on cell lysates and hence, spontaneous complement activation and hydrolysis of C3 in the culture media may cause misleading results regarding C3 localization. Therefore, because of the controversial data on the expression and origin of C3 in B cells, we re-investigated the source and intracellular localization of the protein in human B lymphocytes. Our data show that endogenous expression of C3 is very low in B cells and its main source is indeed the serum. Further, we demonstrate that a part of endocytosed C3 enters the nucleus, binds to the DNA and histones and in such way, might regulate the expression of key genes involved in lymphocyte development and differentiation. Our data reveal a novel, hitherto undefined role of C3 in immune cell homeostasis and regulation of adaptive immunity, which can directly contribute to the better understanding of abnormal B cell responses and treatment of C3-deficient patients.

## Materials and Methods

### Cell Lines, Proteins, Antibodies, and Sera

The human malignant B cell lines (Raji, Namalwa, Ramos, BJAB, Daudi), the Jurkat human T cell line and the monocytoid THP-1 cells, all obtained from ATCC, were grown in RPMI 1,640 medium supplemented with 10% heat inactivated FBS (hiFBS, Hyclone), 100 units/ml penicillin and 100 μg/ml streptomycin (Hyclone) and maintained at 37°C in 5% CO_2_. HEK293 cells and the C3-expressing human epithelial cell line, A549 - both obtained from ATCC - were cultured in DMEM supplemented with 10% hiFBS (Hyclone). Crispr/Cas9 edited, C3 knockout cells were generated and provided by Dr. Ben King (Lund University) and tested by both PCR and Western blot for the absence of C3. Cells used for analysis were between passage 5 and 20 and were *Mycoplasma* free [VenorGEM Classic kit (Minerva Biolabs)]. Antibodies (Abs) used to study C3 expression were the followings: polyclonal goat anti-human C3 (Quidel, #A304) and polyclonal goat anti-human C3 (Calbiochem, #204869). The monoclonal rat anti-human C3d (#HM2198) used in gel shift assays and ELISA experiments was purchased from Hycult. C3a was detected with the polyclonal rabbit anti-C3a antibody (19) from Complement Technologies (#A218, Western blot) or with the monoclonal mouse anti-C3a/C3adesArg Ab from Hycult (#HM2074, gel shift assays and DNA ELISA). DNA was detected by a mouse anti-double stranded DNA Ab from Immunotools (#21227771). Purified human C3 (#A113), C3b (#A114), C3a (#A118), Factor B- (#A335), and C3-depleted sera (#A314) were from Complement Technologies. MBL- (#SER103) and C1q-depleted sera (#A509) were obtained from BioPorto and Quidel, respectively. C3met was prepared by incubation of purified C3 with 100 mM methylamine, pH 8.0–8.5, for 1 h at 37°C and subsequent dialysis against PBS. Proteins were labeled with AlexaFluor 488 following the manufacturer's instructions (Invitrogen). Normal human serum (NHS) pooled from at least 10 donors, was prepared as described (27) according to permit granted by the local ethics committee of Lund.

### Isolation of Human B Lymphocytes

Peripheral blood mononuclear cells (PBMCs) were isolated by Lymphoprep (Stemcell Technologies) density gradient centrifugation from superfluous buffy coat obtained from the Medical Service (Clinical Immunology and Transfusion Medicine, Lund) according to standard procedures (18) and permit granted by the local ethics committee of Lund. B cells were purified by positive selection using the Miltenyi CD19 Microbeads (Miltenyi Biotec), achieving >95% purity of CD19+ B cells as assessed by flow cytometry analysis using fluorescent anti-CD3, anti-CD16, and anti-CD19 antibodies from Immunotools.

### RNA Isolation, RT-PCR, and Real-Time PCR

RNA was extracted from 2 × 10^6^ cells using RNeasy Kit (Qiagen) and 1 μg reverse transcribed to cDNA by Superscript III (Thermo Scientific). C3 mRNA levels were quantified by real-time PCR using primers and FAM-labeled probes from Thermo Scientific (#Hs00163811_m1), according to the manufacturer's instructions. Data were normalized to the housekeeping hypoxanthine guanine phosphoribosyl transferase (HPRT) gene (#Hs99999909_m1) and expression calculated with the 2-dCt method. PCR was performed using the ViiA7 real-time PCR system (Thermo Scientific). The presence of full-length human *C3* in the Raji B cell line and blood B cells was analyzed via conventional PCR using Phusion DNA polymerase (Thermo Scientific) and the following forward (Fw) and reverse (Rv) primers (numbered from canonical ATG start codon): Fw_27 GCTGCTCCTGCTACTAACCC, Fw_2822 CTGTGGCTGTTCGCACCCT, Rv_2918 CTGGTCTCAGACTCGGTGT, Rv_3818 CAAGGCTTGGAACACCATGA and Rv_4973 CATTCTCGAGTCAGTTGGGGCACCCAAAGA. As a positive control, cDNA prepared from total liver tissue RNA (Thermo Scientific) was used. The reaction consisted of incubation at 98°C for 2 min followed by 35 cycles of 98°C for 10 s, 60°C for 15 s and 72°C for 2 min. The amplified products were separated by electrophoresis on a 1% agarose gel containing the SyberSafe DNA dye (Thermo Scientific).

### Cell Lysate Preparation And Fractionation

Cell lysates were prepared by resuspending cell pellets in cell lysis buffer (1% NP-40, 0.05% SDS, in PBS) containing 1X Halt Protease & Phosphatase Inhibitor Cocktail (Thermo Scientific) and incubating for 30 min on ice. The resulting lysates were then centrifuged for 15 min at 15,000 *g*, supernatants collected and stored at −20°C until further use.

Cytoplasmic and membrane fractions of 10^7^ Raji cells were separated with the Mem-PER Plus Membrane Protein Extraction Kit, according to the manufacturers' instructions (Thermo Scientific). Cytoplasmic and soluble nuclear fractions were isolated using the NE-PER Nuclear and Cytoplasmic Extraction Reagents obtained from Thermo Scientific. After separation of the soluble nuclear content, the cell pellet was further purified by digestion with 300 U/sample Micrococcal nuclease (Thermo Scientific) at 37°C for 15 min, centrifuged at 16,000 g for 5 min and supernatant containing the chromatin-associated protein fraction collected.

### SDS-PAGE and Western Blot

The purified cell lysates and fractions were separated by polyacrylamide gel electrophoresis (PAGE) under reducing (R, 25 mM DTT) or non-reducing (NR) conditions and transferred to a PVDF membrane using semi-dry blotting apparatus (BioRad). The membranes were blocked with Quench solution (50 mM Tris–HCl (pH 8.0), 150 mM NaCl, 0.1% Tween 20, 3% fish gelatin, Norland Products) for 1 h at room temperature (RT) and incubated with the primary antibodies overnight at 4°C. Between each of the following steps, the membranes were washed four times with Immunowash (50 mM Tris–HCl (pH 8.0), 150 mM NaCl, 0.1% Tween 20). Proteins were visualized using polyclonal HRP-conjugated antibodies against rabbit, rat, mouse or goat immunoglobulin (Ig)G (DAKO) diluted in Quench. Membranes were developed by the enhanced chemiluminescence (ECL) method (Millipore) and analyzed with the ImageLab software (BioRad). To allow development of both loading controls and C3(a), membranes were cut and incubated separately from each other with primary antibodies (see Supplementary Material). Figures were prepared to publication format with the Photoshop CS4 software.

### C3 Uptake

To analyze endocytosis of C3, 2 × 10^6^ Raji, Jurkat or THP-1 cells were extensively (5X) washed in PBS and thereafter incubated with NHS, purified C3 or C3met or heat inactivated FBS, either in dextrose gelatin veronal buffer (DGVB++) (2.5 mM veronal buffer, pH 7.3, 72 mM NaCl, 140 mM glucose, 0.1% gelatin, 1 mM MgCl_2_, and 0.15 mM CaCl_2_), or Mg-EDTA (2.5 mM veronal buffer, pH 7.3, 72 mM NaCl, 140 mM glucose, 0.1% gelatin, 10 mM EGTA, 7 mM MgCl_2_) or EDTA-GVB (2.5 mM veronal buffer, pH 7.3, 72 mM NaCl, 0.1% gelatin, 140 mM glucose, and 40 mM EDTA) or PBS without calcium and magnesium (Hyclone) for 1 h at 37°C with shaking at 400 rpm. After incubation, cells were washed three times in PBS, pelleted and lysed as described above.

### Confocal Microscopy

To analyze intracellular localization of C3, 2 × 10^6^ Raji B cells were incubated with AlexaFluor 488 labeled C3 in PBS for 1 h at 37°C. After treatment, cells were washed three times with PBS and 3 × 10^5^/sample cytospined (Thermo Scientific) at 86 g for 5 min onto SuperFrostPlus object slides (Menzel, Braunschweig). After fixation with 4% paraformaldehyde for 20 min at 4°C, nuclei were counterstained with mounting medium containing DAPI (Thermo Scientific). Images were obtained with a LSM 700 confocal microscope using a × 63 oil objective and Zen 2009 software (Zeiss).

### Analysis of C3a Generation

To reveal the origin and mechanism of C3a generation in human B cells, 2 × 10^6^ Raji B cells were treated for 1 h at 37°C with 20% NHS in DGVB++ (activation of all pathways), Mg-EGTA (only alternative pathway) or EDTA-GVB buffer (no complement activation). The involvement of complement pathways in activation of C3 was investigated using 20% C1q- (classical pathway), 20% MBL- (lectin pathway) in DGVB++, or 20% FB-depleted serum (alternative pathway) diluted in Mg-EGTA buffer. The involvement of intra- or extracellular enzymes was further analyzed by incubating the cells with 200 μg/mL purified C3 or C3met either in the absence (only intracellular cleavage) or presence (intra- and extracellular cleavage) of 20% C3-depleted serum in Mg-EGTA buffer. After incubation, cells were washed three times in PBS, pelleted and lysed as described above. Lysates were run either under NR (C3) or R (C3a) conditions and C3a generation was followed by Western blot.

### Interactions Between C3/C3a/C3b and DNA—ELISA

Maxisorp microtiter plates were coated with 5 μg/mL genomic DNA (gDNA) isolated from Raji cells (QIAamp DNA Blood Mini Kit, Qiagen) or with 100 and 500 μg/mL C3, or 1 and 5 μg/mL C3a in PBS (Medicago AB) overnight, at 4°C. As a negative control, alpha-1-antitrypsin [A1AT, purified in house from plasma (28)] was used. Between each of the following steps, the plates were washed four times with 0.1% BSA-PBST [0.1% bovine serum albumin (Sigma) in PBS, 0.05% Tween 20 (Fisher Scientific)]. After coating, plates were blocked in 3% BSA-PBST for 1 h at RT and incubated with 2 μg/mL gDNA or 100 μg/mL C3, or 100 μg/mL C3b, or 1 μg/mL C3a for 2 h at RT. After incubation, bound proteins were detected using rat anti-human C3d (C3 and C3b detection) or mouse anti-human C3a/C3adesArg (C3a detection) or with mouse anti-human double stranded DNA antibody (DNA detection). Primary antibodies were diluted 1000X in Quench solution and applied for 1 h at RT. After incubation, HRP-conjugated rabbit anti-mouse or goat anti-mouse immunoglobulins (Igs) were added to the wells (2000X in Quench, Dako). As substrate, 1,2-phenylenediamine dihydrochloride (OPD, Dako) was used and absorbance at 490 nm was measured using a Cary50 MPR microplate reader (Varian).

### Gel Shift Assays

Binding of C3, C3a, and C3b to isolated gDNA of Raji cells, to pCEP4 plasmid and to PCR amplified DNA products was evaluated using gel shift analysis. DNAs and proteins were mixed using 1 μg DNA and 25 μg C3 (5.5 μM), 25 μg C3b (5.5 μM), or 1 μg C3a (5.5 μM) in a 20 μl reaction volume, containing Tris-EDTA buffer (10 mM Tris-HCl, 1 mM EDTA, pH 7.6) and incubated for 30 min at 37°C. As negative control, 25 μg A1AT was used. To analyze whether the interaction is ionic in nature, C3 or C3a and gDNA were incubated in the presence of increasing NaCl concentration (0, 150, 300, 600, 1,200 nM). The presence of C3, C3b, and C3a in the shifted protein-DNA complexes was analyzed via supershift assay, incubating the preformed complexes with 2 μg anti-C3d (C3 and C3b) or anti-C3a/C3adesArg (C3a) Abs for an additional 20 min. As negative controls, purified rat or mouse IgG were used (BD Pharmingen). DNA–protein complexes were separated by 0.8% agarose gel electrophoresis in 40 mM Tris acetate (pH 8) buffer and 0.5 μg/ml ethidium bromide and visualized by UV transluminator.

### Chromatin Immunoprecipitation and DNA Sequencing

To analyze the binding of internalized C3 to DNA, chromatin immunoprecipitation (ChIP) was carried out, as described (29). Briefly, 2 × 10^6^ Raji cells were activated or not with 10 μg/mL CpG ODN2006 (Invivogen) overnight at 37°C before incubation with 200 μg/mL C3. After treatment, cells were extensively washed in PBS, cross-linked by 1% formaldehyde (Thermo Scientific) and sonicated by Bioruptor sonicator (Diagenode) for 25 cycles of 30 s with a 30 s interval (medium intensity) period between cycles, achieving approximately 400–500 bp genomic DNA length after sonication. Lysates were then centrifuged, and the supernatants (sonicated chromatin) were collected. Ten percent volume of each sample was removed as the input control. Sonicated chromatin (30 μg) was incubated overnight at 4°C with 1 μg of anti-C3c antibody (Dako), or a normal rabbit polyclonal IgG (Cell Signaling Technology) as a negative control. Immune complexes were captured with 10 μl of 50% protein A/G coated Dynabeads (Thermo Scientific) and eluted by reverse cross-linking and protease K digestion for 2 h at 68°C. DNA fragments were purified using ChIP DNA clean and concentrator kit (ZymoResearch).

Barcoded DNA adapters were ligated to both ends of the double-stranded cDNA and subjected to PCR amplification. The resultant library was checked on a Bioanalyzer (Agilent) and quantified. The libraries were multiplexed, clustered, and sequenced on an Illumina HiSeq 2000 (TruSeq v3 chemistry). After filtering dirty reads, including low quality reads, N reads and adaptor sequences, clean reads were mapped to reference genome using SOAPaligner/soap2. The identified peaks (submitted to public database ArrayExpress, accession number: E-MTAB-7506) were classified according to their location and function (gene ontology classification). Two of the top genes were selected from CpG-activated samples and quantified by SYBR Green PCR (Applied Biosystems) with primers designed for immunoprecipitated intronic region of *IL-7* and *L-selectin*. Primers were the following: *IL-7* (forward primer AGCTCCCACATACGTCCCAC and reverse GGCAGAAGGCCCTGGTATAG) and *L-selectin* (forward primer GCACAGGGACAAATCTTACACAC and reverse ATTTACCCCATGGAAAGGTGGG). The DNA quantitation value of each sample was analyzed by the 2–ΔCt method and results were calculated by the percent input method using the following formula: [–ΔCt = Ct[IP]-Ct(IC)+Log2(DF) and (2–ΔCt) × 100(%)] (30). In all experiments, we verified that ChIP precipitation enrichment obtained was relative to IgG controls.

### Interaction Between C3/C3a/C3b and Histones—ELISA

Maxisorp microtiter plates were coated with 20 μg/mL human histone H1 (Sigma) or full length histone core octamers (i.e., H2A/H2B/H3/H4, Sigma) in PBS overnight, at 4°C. As negative control, 20 μg/mL A1AT was immobilized. Between each of the following steps, the plates were washed four times with 0.1% BSA-PBST. After coating, plates were blocked in 3% BSA-PBST for 1 h at RT and incubated with distinct concentrations of C3, or C3b, or C3a, or BSA in 0.1% BSA-PBST for 2 h at RT. After incubation, bound proteins were detected using rat anti-human C3d (1,000X in Quench, C3 and C3b detection) or rabbit anti-human C3a (20,000X in Quench, C3a detection), followed by HRP-conjugated rabbit anti-mouse Ig (2,000X in Quench) or goat anti-rabbit Ig (10,000X in Quench, DAKO). As substrate, OPD was used and absorbance at 490 nm was measured as described above.

### Histone H1—C3a Competition

Maxisorp microtiter plates were coated with 10 μg/mL gDNA isolated from Raji cells overnight, at 4°C. As a negative control, A1AT was used. Between each of the following steps, the plates were washed four times with 0.1% BSA-PBST. After coating, plates were blocked with Quench solution for 1 h at RT and incubated with 40 μg/mL AlexaFluor 488 labeled histone H1 in the presence or absence of increasing concentrations of C3a (range: 0–120 μg/mL) for 2 h at RT. As negative control, BSA was applied instead of C3a. After incubation, plates were washed and fluorescence detected using Cytation 5 (BioTek Instruments).

### Statistical Analysis

All data were analyzed using Prism software version 7 (GraphPad). Differences between untreated and C3/C3met treated samples in ChIP experiments were evaluated by 2-way ANOVA with Dunnett's (ChIP ELISA) or with Sidak's (ChIP qPCR) multiple comparison. Binding of C3, C3a, and C3b to histones and competition between C3a and H1 were analyzed using two-way ANOVA with Dunnett's multiple comparison. Differences with *p* < 0.05 were considered statistically significant.

## Results

### Human B Cells Express Very Low Level of C3

**Figure 1:**
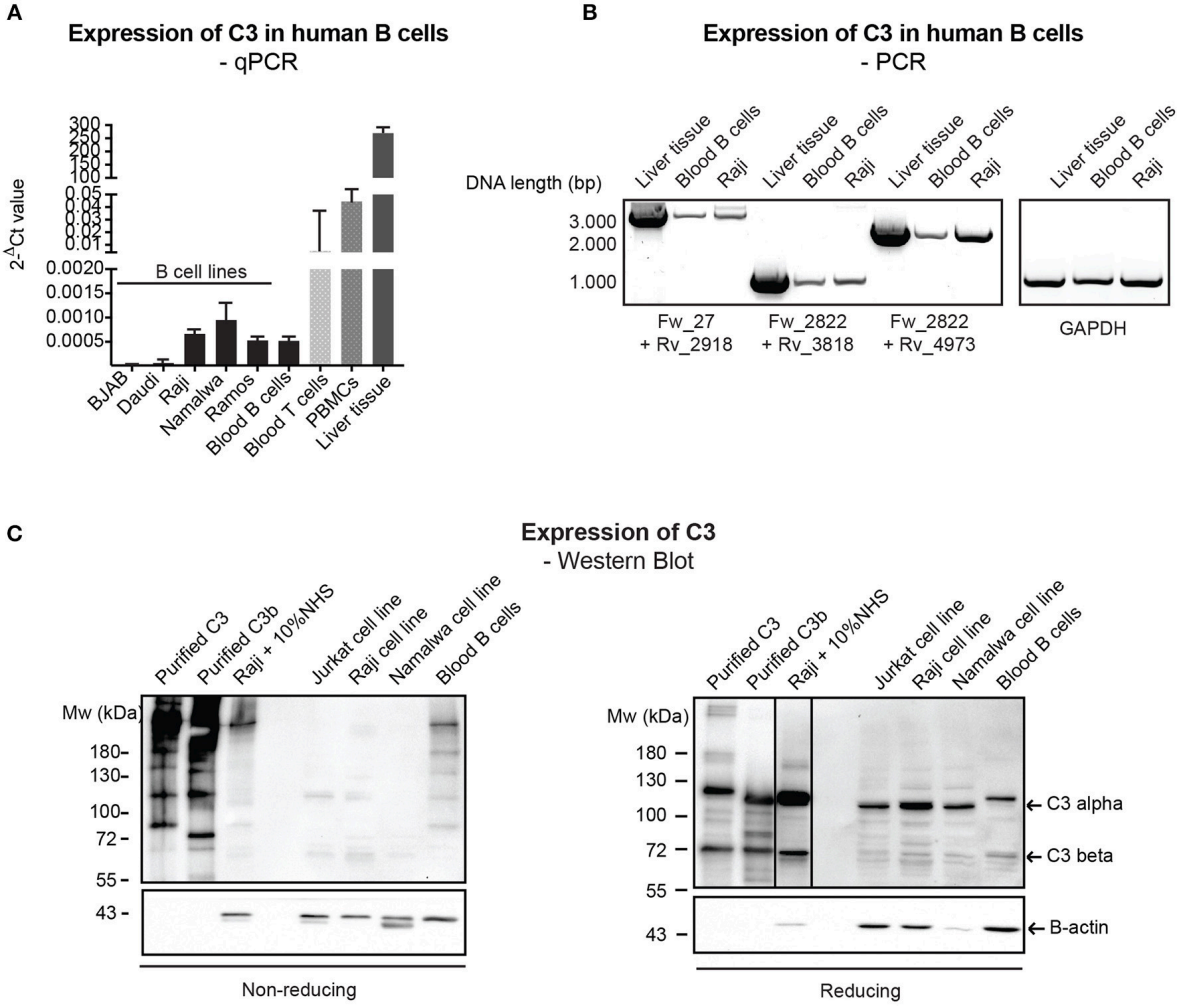
Endogenous expression of C3 is very low in human B cells. **(A)** RNA was isolated from malignant B cell lines and blood-derived B cells, reverse-transcribed and analyzed for C3 expression by qPCR. As positive control, blood-derived T cells, PBMCs, and total, liver tissue RNA were used. Data were normalized to the housekeeping HPRT gene and are shown as mean 2-dCt values with SD of three independent experiments. **(B)** The presence of full length C3 mRNA was confirmed by primer pairs, covering the whole region of human C3 coding sequence. As positive control, liver tissue RNA was used. Data shown are representative of three independent experiments. Numbers indicate DNA length in base pair (bp). The start positions of forward (Fw) and reverse (Rv) primers are shown under the gel picture. **(C)** Western blot results analyzing endogenous C3 expression of human B cells. Lysates prepared from the human B cell lines, Raji and Namalwa and blood-derived B cells and PBMCs were analyzed for the presence of C3 by Western blot with the goat polyclonal anti-C3 antibody from Quidel under non reducing and reducing conditions. As positive control, lysate of Raji cells incubated with 10% NHS in EDTA-GVB buffer was used. Results shown are representative of five independent experiments.

Despite the relatively well-characterized expression of C3 in various immune cells, results regarding the presence of endogenously expressed C3 in human B cells rely mainly on PCR data without clear characterization of C3 expression at the protein level (12, 23). Therefore, firstly we investigated C3 expression in primary, blood derived human B cells and distinct malignant B cell lines by qPCR, semi-quantitative PCR (mRNA level) and Western blot (protein level). qPCR analysis of C3 expression showed that transcription of C3 is very low in human B cells (Figure 1A) in contrast to human T cells and PBMCs, which have been shown earlier to express the protein (12, 13). The presence of full length C3 mRNA in B cells has been confirmed by semi-quantitative PCR, applying primer pairs covering the full length of C3 coding sequence (Figure 1B). Despite the clear evidence of C3 expression at the mRNA level, the 180 kDa mature C3 protein could not be detected under non-reducing (NR) conditions in the malignant B cell lines in contrast to the freshly isolated, blood derived B cells, which may contain serum-derived C3 (Figure 1C, left panel). Although on reducing (R) SDS-PAGE, an abundant C3b-like signal around 110 kDa could be observed in the B cell lines too (Figure 1C, right panel). This signal might be unspecific since it could be detected in Crispr/Cas9 edited, C3 knock out A549 cells (Supplementary Figure 1). Taken together, these results confirm the presence of low levels of mRNA coding for C3 in human B cells, both in primary blood B cells and B cell lines, but the main source of C3 protein in these cells appears to be serum.

### B Cells Actively Take Up C3 From the Extracellular Space

**Figure 2:**
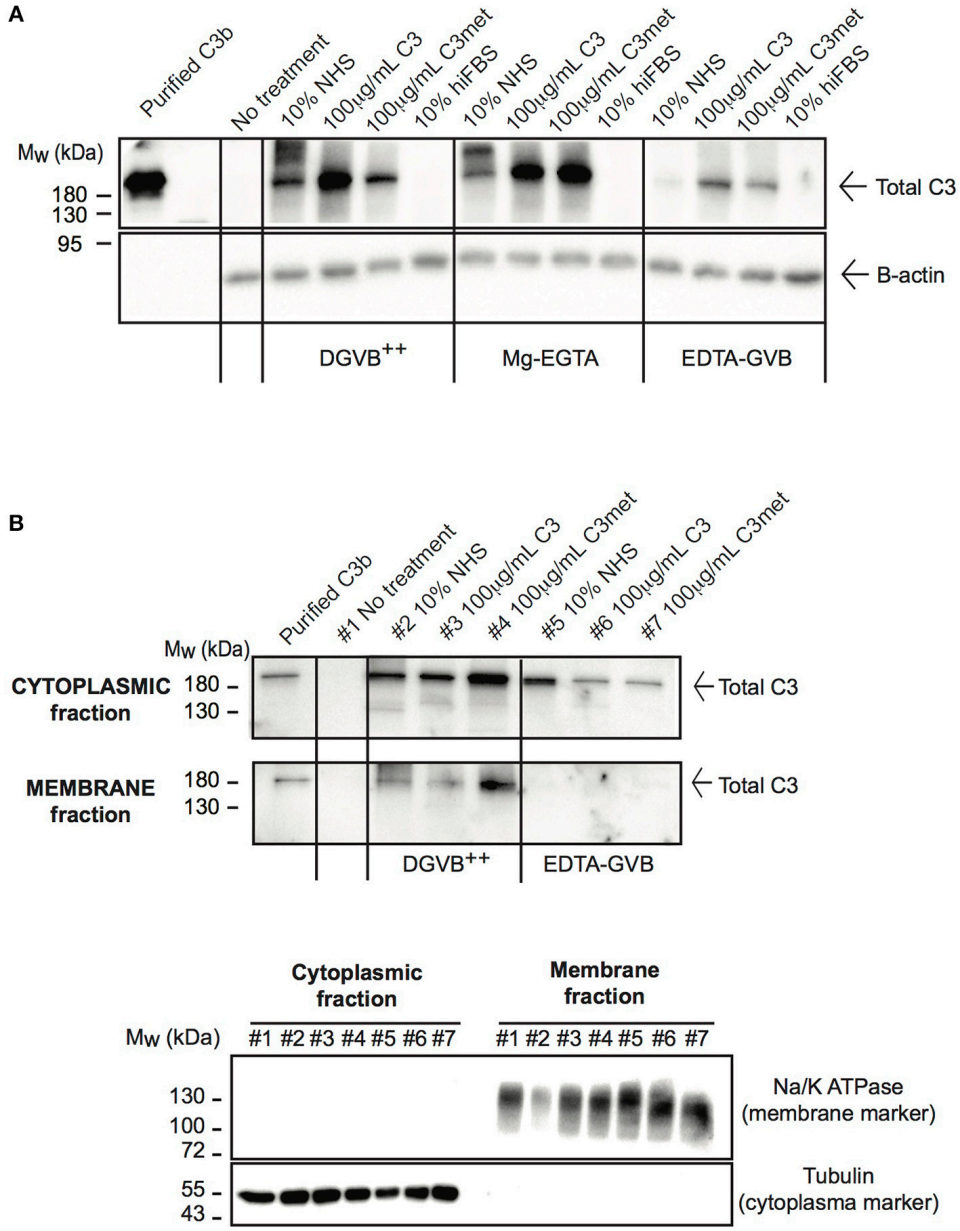
C3 is internalized by human B cells. **(A)** Western blot results analyzing the uptake of C3 by Raji B cells. Cells were treated with distinct sources of C3 (10% NHS, 100 μg/ml C3, 100 μg/ml C3met, 10% hiFBS,) in DGVB++, Mg-EGTA, or EDTA-GVB buffer for 1 h at 37°C. C3 uptake was investigated by Western blot using the goat polyclonal anti-C3 antibody (Quidel) under non reducing conditions. Data shown are representative of four independent experiments. **(B)** Western blot results confirming the presence of internalized C3 in the cytoplasm. Cells were incubated with distinct sources of C3 (20% NHS, 200 μg/ml C3, 200 μg/ml C3met) in DGVB++ or EDTA-GVB buffer for 1 h at 37°C. After lysis, cytoplasmic and membrane fractions were separated and analyzed by Western blot with the goat polyclonal anti-C3 antibody (Quidel) under non reducing conditions. The purity of distinct cellular fractions was verified using antibodies against B-tubulin (cytoplasmic marker) and Na/K ATPase (membrane marker) under reducing conditions. Results shown are representative of two independent analyzes.

Due to the low level of endogenous C3 expression in human B cells and the abundant signal of C3 in freshly isolated B cells (Figure 1C), the main source of C3 observed in B cells is likely the extracellular space as suggested previously (23). Nevertheless, alternative pathway may be activated on the surface of B cells, leading to deposition of C3 on the plasma membrane (26, 31). Hence a strict control of experimental conditions is necessary to selectively measure the uptake of C3 and exclude the detection of membrane-bound forms of the protein. Due to these reasons we re-investigated C3 uptake by human B cells under specific conditions controlling the degree of possible complement activation and from different sources of the protein. The malignant human B cell line, Raji was treated either with NHS, purified C3, methylamine treated C3 (C3met) or with heat inactivated fetal bovine serum (hiFBS), a commonly used supplement of cell culture media. FBS was included in our analysis since many of the antibodies, which react with human C3, also recognize bovine C3 (data not shown). C3 uptake was investigated in DGVB++ buffer (allows activation of all complement pathways and hence C3 cleavage and membrane binding/deposition), in Mg-EGTA buffer allowing only alternative pathway activation or in EDTA-GVB buffer in which complement activation is blocked and hence, no C3 activation and surface deposition may occur. As illustrated in Figure 2A, the most abundant C3 signal was observed in the cells in DGVB++ buffer. Inhibition of complement activation in EDTA-GVB buffer resulted in decreased but detectable C3 signal in Raji cells incubated either with NHS or the purified proteins including C3met, which can no longer be activated by complement convertases and resembles C3b in structure. These results were confirmed using the human leukemic T cell line, Jurkat and the monocytoid THP-1 cells, indicating that the uptake of C3 from the extracellular space occurs in various cell types (Supplementary Figure 2). Despite the noticed cross-reaction of anti-human C3 Abs with bovine C3, the protein could not be detected in the cells after incubation with hiFBS suggesting that intracellular C3 does not derive from this medium supplement.

To selectively measure C3 uptake and exclude surface binding, membrane and cytosolic fractions of NHS-, C3-, and C3met-treatedRaji cells were separated and analyzed for the presence of C3 (Figure 2B). We found that in DGVB++ buffer, which contains divalent cations, C3 could be detected both in the cytoplasm and in the membrane fraction. However, in EDTA-GVB buffer, C3 was observed only in the cytoplasmic fraction, indicating that C3 signal observed in the cells in EDTA-GVB buffer (Figure 2A) derives from internalized C3.

Taken together, these data together with Western blot results of Figure 2A prove that both C3 and C3met are efficiently internalized by human B cells from the extracellular milieu.

### C3a Is Present in Human B Cells and Is Generated by Alternative Pathway C3-Convertases

**Figure 3:**
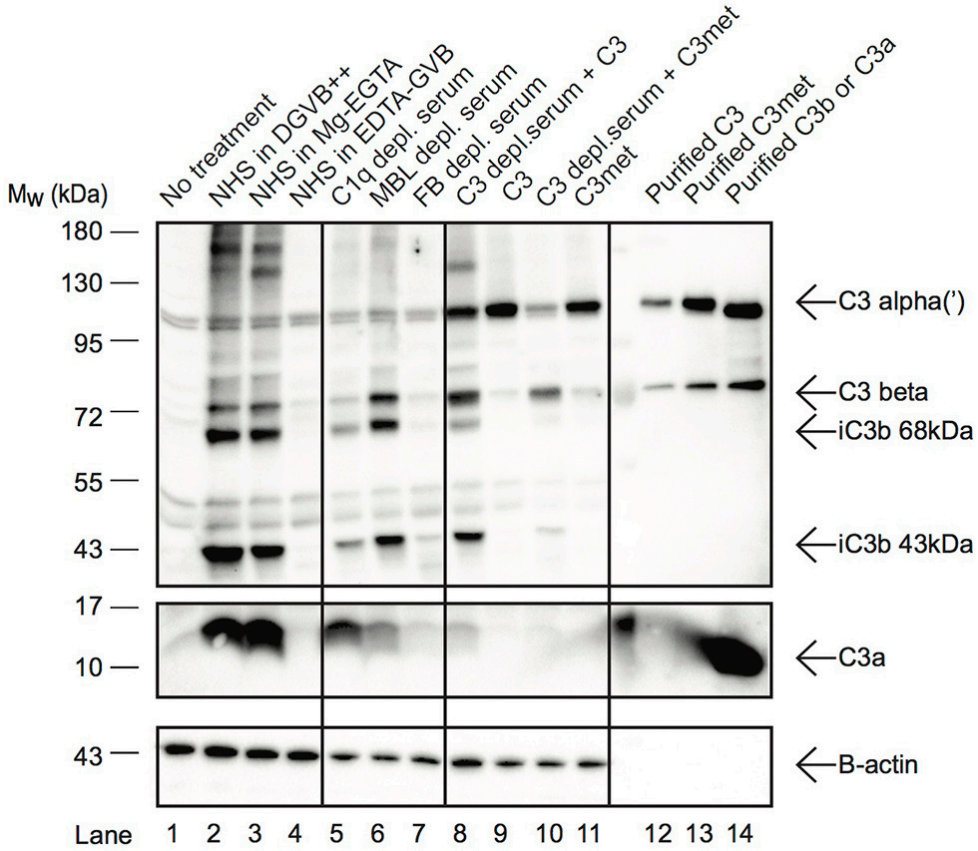
C3a in B cells is generated extracellularly by alternative pathway C3-convertases. Western blot results investigating the origin of C3a in Raji B cells. Cells were incubated with distinct sources of C3 (10% NHS, 100 μg/ml C3, 100 μg/ml C3met) in DGVB++, Mg-EGTA, or EDTA-GVB buffer for 1 h at 37 °C. To analyze complement pathway dependent generation of C3a, sera depleted in C1q (classical pathway), MBL (lectin pathway) or Factor B (alternative pathway) were used. To distinguish intracellular or C3-convertase dependent processing of C3, C3, and C3met were added alone (intracellular cleavage) or in the presence of C3-depleted serum (C3 convertase supplementation). C3 processing and C3a generation were analyzed by Western blot with the goat polyclonal anti-C3 (Quidel) and rabbit anti-C3a (Complement Technologies) antibodies under reducing conditions. Result shown is one representative out of three independent experiments.

In resting T cells, intracellular C3 is continuously processed into C3a and C3b, transducing homeostatic survival signals via mTOR activation (12). Although cleavage of C3a from endocytosed C3 by intracellular proteases has been indicated in B cells too (23), interestingly we could not detect C3a in Raji B cells after treatment with NHS in EDTA-GVB (Figure 3). To clarify the origin of C3a in B cells, Raji cells were treated either with purified C3, C3met or with NHS in different buffers, allowing activation of either all (DGVB++) or only the alternative (Mg-EGTA) complement pathways. As shown in Figure 3, C3a could be detected in B cells when the cells were incubated with NHS either in DGVB++ (lane 2) or in Mg-EGTA (lane 3) buffer, however the signal almost completely vanished when complement activation was blocked (lane 4, EDTA-GVB buffer). In accordance with these results, C3a could not be observed in the cells after incubation with purified C3 (lane 9) or C3met (lane 11), further supporting that C3a is generated by a serum-derived C3-convertase on B cells and not by intracellular proteases. Indeed, supplementation of purified C3 with serum C3-convertases (i.e., addition of C3-depleted serum in parallel with purified C3) restored C3a level in the cells (lane 8). This effect is not apparent in case of C3met, which cannot be cleaved by serum convertases (lane 10). Since in Mg-EGTA buffer (lane 2) less C3a was generated in comparison with DGVB++ buffer (lane 1), we analyzed which complement pathway is involved in C3a production. To this end, sera depleted of specific components of either the classical (C1q), lectin (MBL), or alternative (Factor B) pathways were used and generation of C3a monitored. Almost no C3a was present when Factor B depleted serum was used as source of C3 (Figure 3, lane 7). Depletion of C1q (lane 5) and MBL (lane 6) resulted in only minor changes in C3a level. In summary, these results show that C3a—in contrast to T cells and earlier observations in B cells—is generated by the alternative pathway C3 convertases in human B cells.

### C3 Enters the Nucleus After Endocytosis

**Figure 4:**
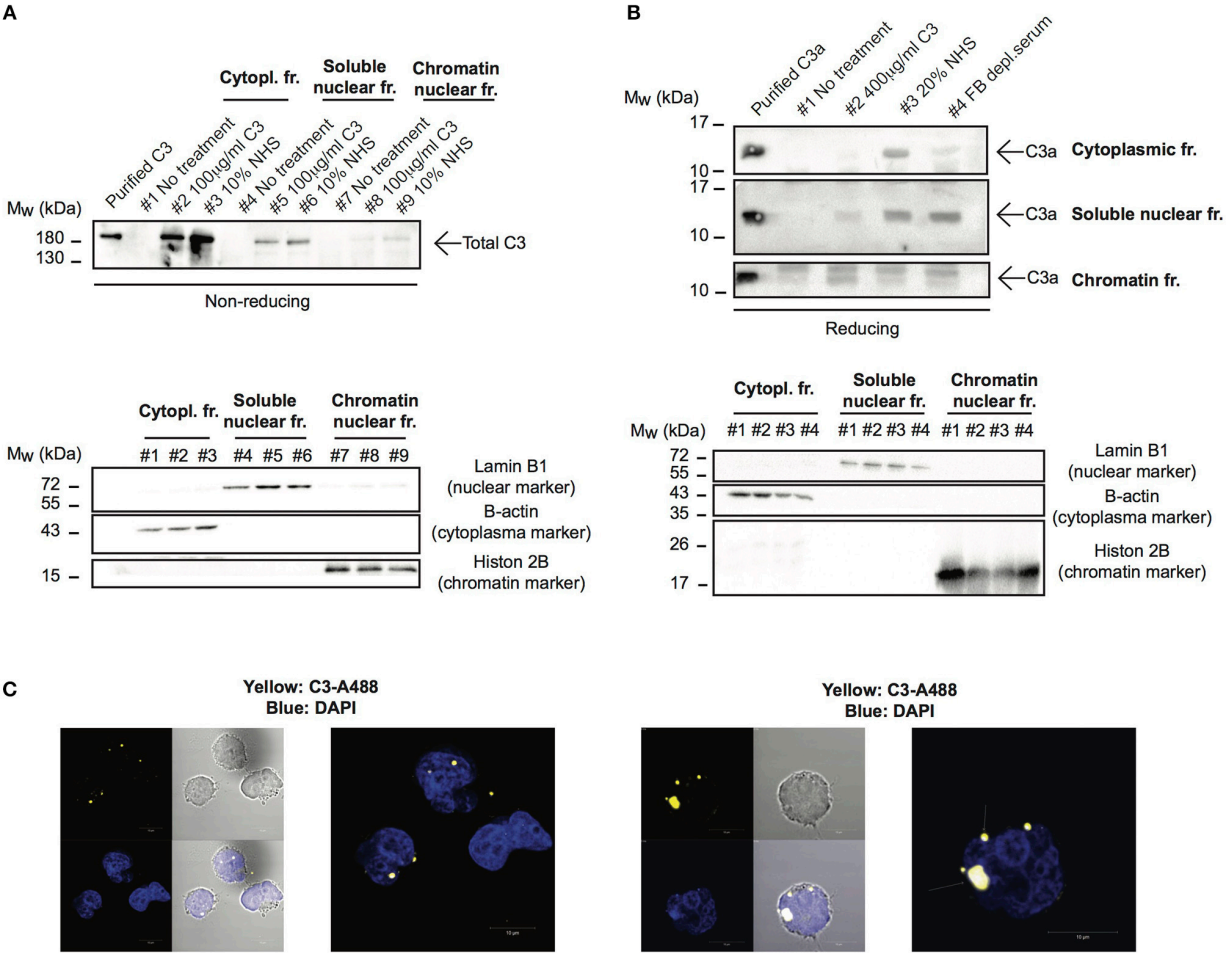
C3 and C3a enter the nucleus after uptake. **(A,B)** Western blot results showing presence of C3 and C3a in nuclear compartments of Raji cells. The human B cell line, Raji was incubated with NHS, C3 or C3met as a source of C3 either in EDTA-GVB **(A)** or in Mg-EGTA **(B)** buffer for 1 h at 37°C. After lysis, cytoplasmic, soluble nuclear, and chromatin-associated nuclear fractions were separated and analyzed by Western blot with the goat polyclonal anti-C3 antibody under non reducing conditions **(A)** or with the rabbit polyclonal antibody against C3a under reducing condition **(B)**. The purity of distinct cellular fractions was verified using antibodies against B-actin (cytoplasmic marker), lamin B1 (nuclear marker) and histone H2B (chromatin-associated nuclear marker). Results shown are one representative experiment out of three **(A)** or four **(B)** independent analyzes. **(C)** Representative confocal images showing that AlexaFluor 488 labeled C3 enters the nucleus. 2 × 10^6^ Raji cells were incubated with 100 μg/ml C3-AlexaFluor 488 for 30 min at 37°C, fixed and counterstained with DAPI using mounting medium. Representative images are shown from two independent experiments investigating at least 50 cells/analysis.

Although C3 uptake by leukocytes has been published, its intracellular localization has not been followed earlier. Since localization of the protein strongly influences its function and interacting partners, we investigated which cell compartments that contain C3 after internalization. To this end, cells were treated with different sources of C3 either in EDTA-GVB (Figure 4A) or in Mg-EGTA (Figure 4B) buffers, fractionated into cytoplasmic, nuclear and chromatin-associated fractions and analyzed by Western blot for the presence of C3 (Figure 4A) or C3a (Figure 4B). We found that the major destination of extracellular-derived C3 is the cytoplasm but the protein could also be detected in the soluble nuclear fraction of Raji cells. Furthermore, C3 was observed in the chromatin-associated cellular fraction, indicating its interaction with the nuclear material. Similar to C3, C3a was also transported to the nucleus (Figure 4B). Nuclear entry of C3 was confirmed by confocal imaging: the fluorescently labeled and internalized C3 was present in DAPI labeled nuclei of B cells (Figure 4C). Taken together, these data indicate that a portion of extracellular, serum-derived C3 enters the nucleus after endocytosis.

### C3 Interacts With DNA

**Figure 5:**
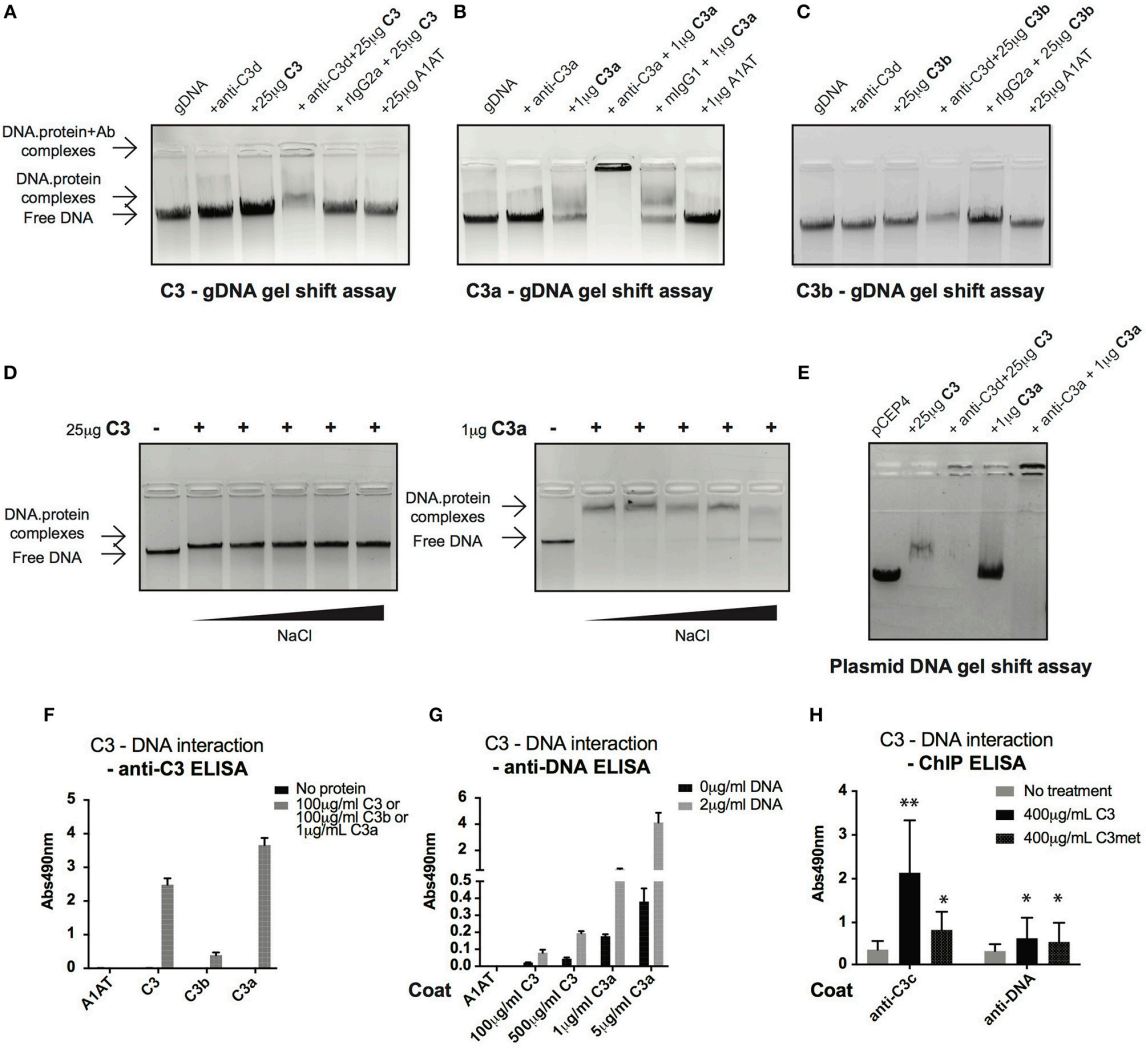
C3 and C3a bind to distinct types of DNA. **(A–C)** Gel shift assays presenting interaction between C3(a) and genomic DNA isolated from Raji cells. Isolated genomic DNA was incubated either with pre-titrated concentrations of C3 **(A)** or C3a **(B)** or C3b **(C)** and separated by agarose gel electrophoresis. Formation of high molecular weight DNA-protein complexes is indicated by slower DNA migration (DNA shift) compared to migration of the free nucleic acid. The presence of C3 and its cleavage fragments in the pre-formed protein-DNA complexes was confirmed with anti-C3a and anti-C3d antibodies caused supershift. One representative experiment out of five performed is shown. **(D)** Gel shift assays showing that C3(a)—DNA interaction is not ionic in nature. C3 or C3a were incubated with genomic DNA of Raji cells in the presence of increasing NaCl concentration (ranging from 150 to 1,200 mM). Binding of C3 and C3a to DNA was observed at 8X higher salt concentration than the physiological 150 mM. **(E)** Interaction of C3 and C3a with DNA is not restricted to genomic DNA. Linearized pCEP4 vector was incubated with either C3 or C3a and DNA-protein complex formation was investigated by gel shift assay. Results illustrated are one representative out of three independent experiments. **(F,G)** ELISA results confirming interaction between C3(a) and DNA. ELISA microplate surfaces were coated either with DNA (F) or C3, C3a, C3b **(G)**. After incubation with distinct concentrations of C3 proteins **(F)** or Raji genomic DNA **(G)**, protein-DNA interactions were detected using anti-C3d, anti-C3a (anti-C3 ELISA, **F**), or anti-dsDNA antibody (anti-DNA ELISA, **G**). Results shown are mean absorbance values measured at 490 nm with SD of four independent experiments. **(H)** ELISA results showing presence of C3-DNA complexes in formaldehyde cross-linked chromatin fraction of Raji B cells. Cells were incubated in the presence or absence of C3, C3met, fixed with 1% formaldehyde to cross-link protein-DNA complexes and chromatin isolated using commercial kit. C3-DNA complexes were investigated by ELISA on anti-C3 or anti-DNA coated plates. Data are shown as mean absorbance values measured at 490 nm with SD of three independent experiments. Differences with *p* < 0.05 were considered statistically significant and compared to non-treated cells (two-way ANOVA with Dunnett's multiple comparison, ^ns^*p* > 0.05, ^*^p < 0.05, ^**^p < 0.01).

Cell fractionation and confocal imaging data showed that C3 and C3a are present in the nucleus, suggesting their potential interaction with DNA. Therefore, we tested the ability of these proteins to bind to genomic DNA (gDNA) of Raji B cells using gel shift assays. Incubation of gDNA with C3 resulted in a small but significant retention of DNA, indicating the presence of high molecular weight complexes and hence, the binding of C3 to the nucleic acid (Figure 5A). The presence of C3 in the complex was proved by addition of the anti-C3d antibody, which caused decreased mobility (i.e., supershift) of the pre-formed C3-DNA complexes. Neither the negative control, A1AT nor the isotype control, rat IgG2a had any effect on DNA mobility. Similar to C3, incubation of gDNA with C3a resulted in the shift of nucleic acid mobility (Figure 5B). The interaction between C3a and the DNA could be ascertained due to the fact that the anti-C3a antibody caused a supershift of the complexes. In contrast to this, C3b had only negligible effect on the nucleic acid mobility indicating that mainly the C3a part of the molecule interacts with the DNA (Figure 5C). Results of gel shift assays were confirmed using sandwich ELISA (Figures 5F,G): strong interaction could be observed between C3, C3a, and DNA both with gDNA and protein coated surfaces in contrast to C3b which did not bind to the nucleic acid.

C3a is a cationic protein, which could lead to its “unspecific,” ionic interaction with the negatively charged surfaces of DNA. To identify the type of interaction between C3(a) and the nucleic acid, gDNA of Raji cells was incubated with C3 or C3a in the presence of increasing concentrations of NaCl (Figure 5D). The interaction of C3 with DNA was not affected even at the highest salt concentration (1,200 mM). Similarly, C3a-DNA complexes dissociated only at very high NaCl concentration, indicating the specific, non-ionic interaction between the proteins and nucleic acid. To test whether C3 and C3a interact with other types of DNA or if it is specific for the eukaryotic nucleic acid, the proteins were incubated with plasmid DNA. Similar to Raji cell derived DNA, plasmid DNA migrated at higher molecular weight in the presence of C3 and C3a (Figure 5E). The results above indicate a strong interaction between C3(a) and DNA but only using purified components *in vitro*. To investigate whether extracellular C3, after internalization binds to DNA, Raji cells were incubated with C3 or C3met, cross-linked by formaldehyde and the isolated chromatin tested for the presence of C3-DNA complexes by ELISA. In accordance with results of the *in vitro* assays, C3-DNA complexes were present in the chromatin fraction of Raji cells (Figure 5H), supporting the entry of extracellular C3 into the nucleus and its binding to the DNA *ex vivo*.

### C3 Binds to Multiple Genomic Regions

**Figure 6:**
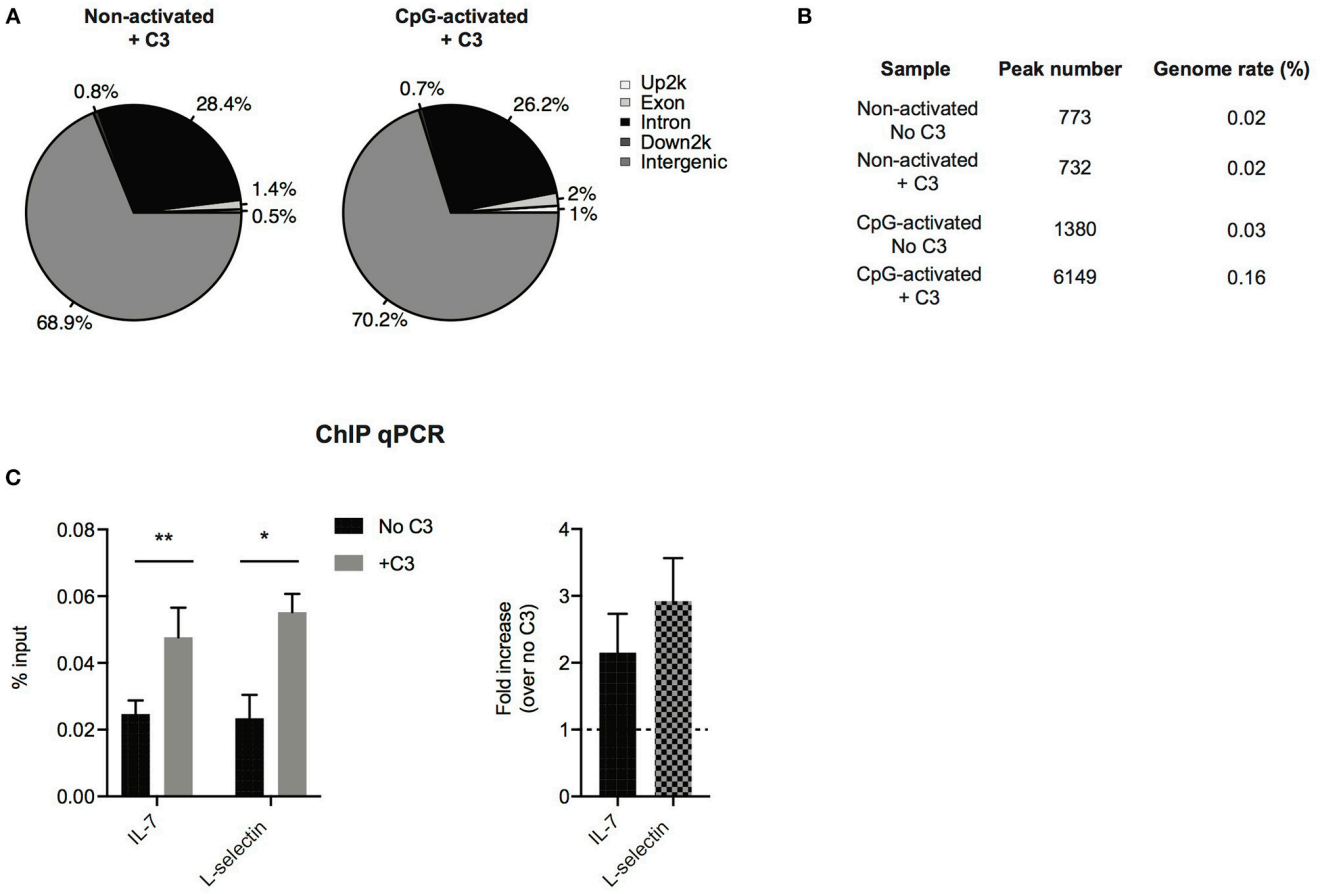
Confirmation of C3-DNA interaction by ChIP sequencing. **(A)** ChIP-seq identified peaks from anti-C3 immunoprecipitated chromatin samples were classified based on the location (UCSC annotation data) and are shown in the following genome regions: intergenic, introns, downstream 5 Kbp, upstream 5 Kbp, and exons. The interacting DNA elements localized mainly the intergenic and intronic regions. **(B)** ChIP-seq statistics of identified peaks including peak numbers and genome rate (%). C3 binding to genomic DNA is strongly increased upon stimulation of B cells with the polyclonal activator, CpG oligonucleotide. **(C)** ChIP-PCR was run on ChIP DNA of CpG-activated samples using primers specific for the ChIP-seq identified regions in *IL-7* and *L-selectin* genes. Enrichment of the specific DNA loci was calculated with the percent input method and illustrated as % input or as fold changes over samples without C3 treatment (two-way ANOVA with Sidak's multiple comparison, ^*^p < 0.05, ^**^p < 0.01). The values represent the mean ± SEM of four independent experiments.

To analyze which genes C3 might interact with and regulate, ChIP sequencing was carried out. To this end, Raji cells were left untreated or stimulated with the polyclonal B cell activator CpG, to induce chromatin remodeling, which renders DNA more accessible to the interacting proteins. Cells were fixed by formaldehyde and chromatin was sheared by ultrasound. Chromatin regions bound by C3 were immunoprecipitated by antibody binding to C3. DNA fragments were isolated and sequenced genome-wide. ChIP-seq analysis confirmed interaction between C3 and genomic DNA, which is mainly located in intergenic and intronic regions (Figure 6A). Compared to untreated cells, the B cell activator CpG strongly stimulated C3-DNA binding, and increased the number of ChIP peaks from 732 to 6,149 (Figure 6B). Pathway analysis of C3-interacting genomic regions identified genes encoding basic transcription factors (CREB5, estrogen receptor 1), proteins involved in calcium signaling and neuronal growth (calmodulin), and also cytokines (IL-7) and signaling molecules (Syk, Zap-70), which are key components in lymphocyte development and differentiation. C3 deficient patients have been previously reported to have abnormal immune response (6). Here two identified genes IL-7 and L-selectin, which are involved in lymphocyte development and activation, were further validated and confirmed by ChIP-qPCR (Figure 6C), suggesting the potential involvement of serum-derived C3 in regulation of lymphocyte maturation.

### C3 Interacts With Histones and Might Induce Chromatin Remodeling

**Figure 7:**
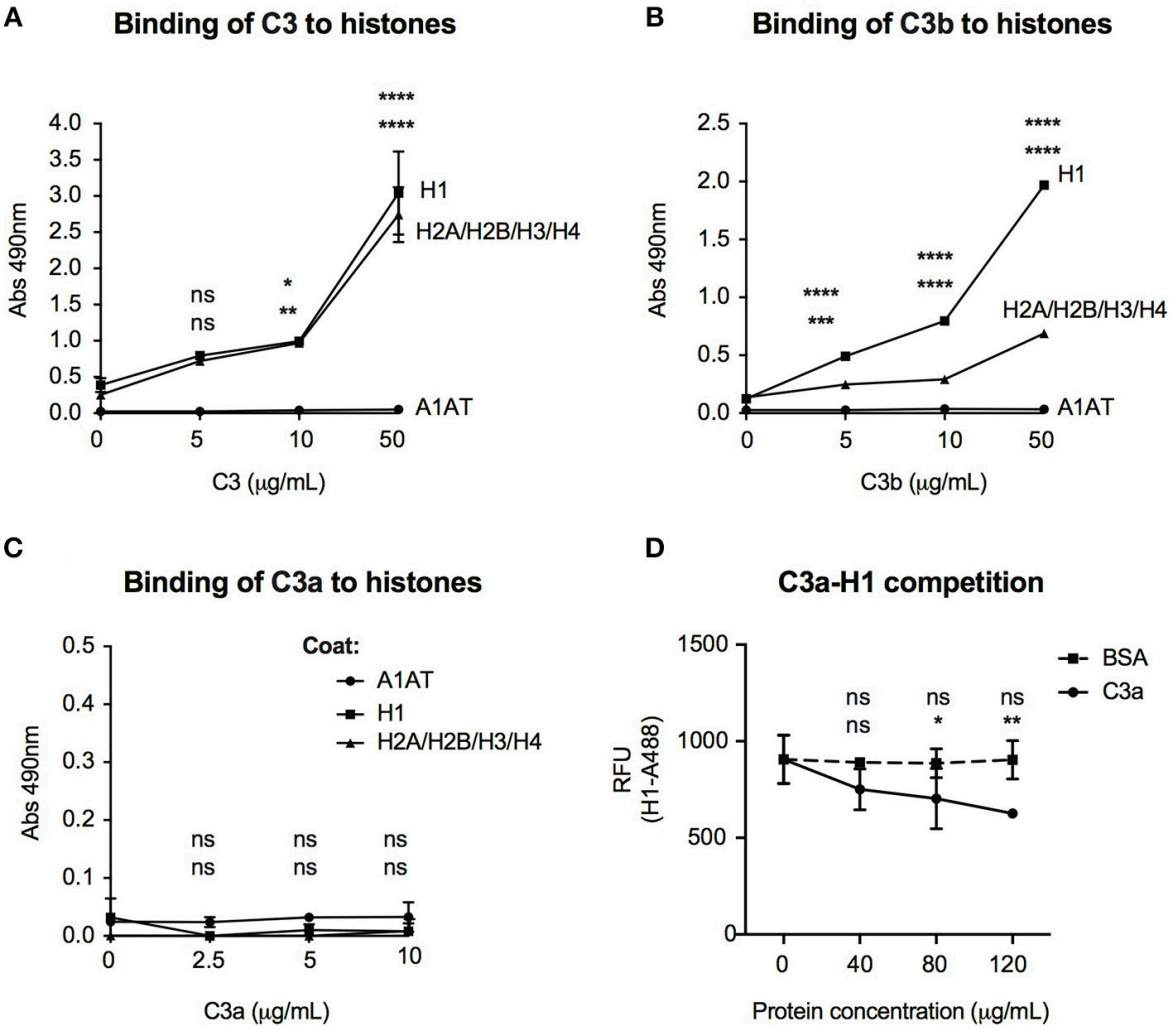
Interaction of C3 and its cleavage fragments with histones. Recombinant histone core octamers (H2A/H2B/H3/H4), the linker H1 histone, or alpha-1-antitrypsin (negative control) were immobilized on microtiter plates and incubated with increasing concentrations of C3 **(A)**, C3b **(B)**, or C3a **(C)**. As negative control, BSA was used. Binding was detected either with monoclonal anti-C3d **(A,B)** or polyclonal anti-C3a **(C)** antibodies. Background signals obtained from BSA incubation were subtracted from the original values. Data are presented as mean absorbance values measured at 490 nm with SD of three independent experiments. Differences with *p* < 0.05 were considered statistically significant and compared to 0 μg/ml protein incubated surfaces (two-way ANOVA with Dunnett's multiple comparison, ^ns^*p* > 0.05, ^*^p < 0.05, ^**^p < 0.01, ^***^p < 0.001, ^****^p < 0.0001). Upper asterisks indicate statistical significance of H1-, lower asterisks of histone octamer coated surfaces compared to binding to A1AT. **(D)** gDNA of Raji cells was coated on microtiter plates and AlexaFluor 488-labeled H1 histone binding was monitored in the presence or absence of C3a. As negative control, BSA was used. Background signals obtained on A1AT coated surfaces were subtracted from the original values. Data are presented as relative fluorescent unit (RFU) measured at 525 nm with SD of two independent experiments. Differences with *p* < 0.05 were considered statistically significant and compared to no C3a treated samples (two-way ANOVA with Dunnett's multiple comparison, ^ns^*p* > 0.05, ^*^p < 0.05, ^**^p < 0.01). Upper and lower asterisks indicate statistical significance of H1-AlexaFluor 488 binding in the presence of BSA or C3a (respectively) compared to binding of H1-AlexaFluor 488 alone.

Histones are basic proteins, which associate with DNA in the nucleus to form chromatin. Changes either in the structure or function of histones induce chromatin remodeling, altering the DNA accessibility for transcriptional factors and hence regulate gene transcription. Since C3 bound mainly to intergenic and intronic regions of DNA (Figure 6A), we investigated whether it might bind to histones and hence, influence chromatin structure. As illustrated in Figure 7A, dose-dependent binding of C3 was observed both to histone H1 (linker histone) and to histone core complex (H2A/H2B/H3/H4) coated surfaces. Interestingly, C3a and C3b showed opposite tendency in histone binding than to DNA: although C3b bound strongly to both H1 and core histones (Figure 7B), C3a did not interact with these proteins (Figure 7C). To investigate the functional consequence of C3-histone interaction, we investigated whether C3 might induce chromatin rearrangement—a potential mechanism leading to altered gene accessibility and transcriptional activity. To this end, two approaches were used. To analyze the direct effect of C3 on the nucleosome structure, a FRET-based chromatin remodeling assay was carried out. Cy3/Cy5 conjugated nucleosomes were incubated in the presence or absence of C3 or its cleavage fragments, and alteration in the chromatin structure was followed via changes in Cy3/Cy5 fluorescence. Further, we investigated whether C3a might interfere with the H1 linker histone in binding to DNA and hence indirectly regulate the transduction between eu- and heterochromatin states. Although in the FRET-based assay we could not observe a direct effect of C3 on chromatin remodeling (data not shown), C3a dose-dependently inhibited histone H1 binding to the DNA (Figure 7D), indicating competition between the cationic peptides.

## Discussion

For a long time, complement activation has been thought to take place almost exclusively in the blood and its components synthesized by the liver and restricted cell types. With the discovery of intracellular complement proteins in immune cells, this classical paradigm has changed over the recent years. It is well-established now that many immune cells are capable of active synthesis of complement proteins and this locally operating intracellular “complosome” is key in driving basic cellular processes and providing immune homeostasis. In this study, we clarified the origin and mechanism of C3 activation in B cells and revealed a novel function of the protein via its nuclear and chromatin binding capacity.

Intracellular C3 may be taken up from the serum (23) or actively synthesized by immune cells (32), indicating that intracellular C3 may have two sources. Intracellular C3 was first demonstrated in freshly isolated human B cells by confocal microscopy (12) and the presence of C3 in primary B cells was later confirmed. However, the results indicated that the main source of C3 observed in B cells is the serum and hence, malignant B cell lines in culture do not contain intracellular stores of the protein (23). Via PCR and qPCR, we now proved low but significant expression of C3 mRNA in both primary, blood-derived B cells and malignant B cell lines (Figures 1A,B). To our knowledge, our data are the first proving endogenous expression of full length C3 mRNA in human B cells. Earlier only an 1.9 kbp mRNA transcript of C3 have been reported in Raji B cells (33). The alternative transcription originated from an internal promoter in intron 8 and generated a 538 amino acids long protein, identical to the C-terminal region of human C3. Although our results indicate that the full length C3 mRNA is present in human B cells (Figure 1B), interestingly upon non-reducing SDS-PAGE—similar to earlier observations (23)—the expected 180 kDa C3 band was missing from the malignant cell lines in contrast to freshly isolated B cells, which may contain serum-derived C3 (Figure 1C). Only a C3b-like alpha chain could be observed in the B cell lines on both reducing and non-reducing SDS-PAGE (Figure 1C). Although this product was recognized by several different antibodies against C3 (Quidel #A304 (Figure 1C), Complement Technology #A213, Sigma #C7761, DAKO #A0062 and #A0063), it is probably unspecific since it can be detected in the Crispr/Cas9 edited, C3 knock out A549 cells and mock transfected HEK293 cells (Supplementary Figure 1). These results indicate that even if C3 mRNA is present in human B cells, its protein level is kept very low probably due to posttranscriptional regulation, and the protein itself is undetectable. Therefore, our results warn for the careful selection of anti-C3 antibodies to investigate the protein's expression and the combined analysis of both mRNA and protein levels in the future to report C3 expression.

Malignant B cell lines, in contrast to freshly isolated, blood derived cells, do not contain C3 signal [Figure 1C and Supplementary Figure 1 and (23)]. Since one of the major differences between cell lines and freshly purified cells is the time of culture in absence of human serum, it was hypothesized that the major source of intracellular C3 is the serum and the cells actively internalize the protein. However, the membrane-bound and cytoplasmic C3 cannot be distinguished via conventional Western blot and hence, spontaneous complement activation and subsequent deposition of activated C3 fragments on the cell surface may confound results. Our data indicate that B cells are indeed able to take up C3 from the extracellular environment, however its uptake can be selectively investigated only in buffers, which block complement activation (Figures 2A,B). C3 uptake was observed in both purified form (both native and methylamine treated C3) and also from serum. However, FBS—a common supplement of traditional media—does not serve as a source of human C3 (Figure 2A), ruling out that the C3 signal in the cells derives from bovine C3, which many antibodies against human C3 cross-react with (personal observation). Although the highest C3 signal in the cells could be observed after incubation with C3 sources in DGVB++ buffer, most of this signal derives from membrane bound fraction of the protein (Figure 2B) and hence, represent deposited, non-internalized C3. The same conclusion was drawn when C3 uptake was investigated in PBS or commonly used culture media, such as RPMI-1640, which all contain divalent cations, necessary for complement activation. Selective internalization of C3 could be investigated only in EDTA-GVB buffer in which activation of all complement pathways are blocked: in this case, C3 could be detected only in the cytoplasmic, but not the membrane fraction of the cells (Figure 2B), indicating clear uptake of the protein. Nevertheless, it is important to note that the exact mechanism of C3 uptake has not been clarified yet (23). Hence, the presence of C3 in the cytoplasm but not in the membrane fraction of the cells does not rule out the possibility that a part of the internalized protein originates from the endocytosis of receptor- or covalently bound membrane form of C3 as part of endosomes and not soluble C3 from the extracellular space. The exact mechanism of C3 uptake is currently being investigated in our group.

Interestingly, when activation of internalized C3 was followed via monitoring C3a generation, we could not detect the anaphylatoxin when C3 sources were provided in EDTA-GVB buffer (Figure 3). These results indicated that C3a, observed in the cells after C3 uptake, is generated extracellularly by serum convertases. Indeed, depletion of Ca2+ and Mg2+ ions almost completely abolished C3a signal in the cells supporting that complement activation is necessary to produce C3a in the cell lines. The cleavage was mediated mainly via the alternative pathway, since depletion of either Factor B or Mg2+ ions strongly decreased C3a generation in contrast to the absence of C1q (classical pathway) or MBL (lectin pathway) or Ca2+ ions (classical and lectin pathway). The extracellular processing of C3 is further supported by the observation that C3a does not appear in the cell after incubation with purified C3 as opposed to the intracellular C3 processing by proteases. Further, supplementation of serum C3 convertases via incubation of cells with purified C3 in the presence of C3-depleted serum led to the appearance of C3a, proving the C3-convertase dependent origin of C3a. These results together confirm that the main source of intracellular C3 is the serum in the malignant cell lines but in contrast to the previous report (23), C3a is mainly generated extracellularly by serum C3 convertases and not by intracellular proteases. As indicated above, the discrepancy between our and the published results is probably caused by the buffers used to investigate C3 uptake since even low concentrations of divalent cations may lead to complement activation and hence, assembly of C3 convertases.

Despite the clear evidence of C3 uptake from the extracellular milieu, its intracellular route has not been followed earlier. Fractionation of C3 incubated Raji cells showed that endocytosed C3 enters the nucleus (Figures 4A,C). Similarly, C3a could also be observed in the nuclear and chromatin-associated fractions of Raji cells (Figure 4B), indicating the potential binding of C3 and C3a to DNA. Indeed, a strong and non-ionic interaction between C3/C3a and the nucleic acid was observed *in vitro* via gel shift assays (Figures 5A–D), ELISAs (Figures 5F,G) and *ex vivo* by chromatin isolation and immunoprecipitation (Figures 5H,6 respectively). The interaction was mediated mainly via the C3a part of the protein since C3b did not induce DNA shift (Figure 5C). Interestingly, C3 and C3a bound not only to Raji derived genomic DNA but also to bacterial plasmids (Figure 5E). Although our study focused on eukaryotic DNA, the binding of C3 to prokaryotic nucleic acids suggest that the interaction may have multiple roles depending on the organism. For example, non-enveloped viruses or bacteria, which usually avoid extracellular complement attack, could perhaps be opsonized intracellularly by C3, aiding the clearance of the invasive pathogens. This hypothesis is supported by two recent reports, demonstrating C3-mediated restriction of viral and bacterial growth via autophagy- and proteasome targeted degradation of the target structures (19, 34). Additionally, C3a is a strong antimicrobial peptide acting via disruption of bacterial membrane integrity (35). Similar to C3a, many antimicrobial peptides (NK-18, hLF1-11 or P-113) have been shown to be actively taken up by bacteria and bind to DNA. These data indicate that C3a may penetrate the prokaryotic cell membrane and its antimicrobial effect may involve DNA binding. Although the DNA binding property of C3a in clearance of infections requires further investigation, it may directly support the recent view of complement evolution suggesting that the original role of C3 uptake is to mediate a rapid response of the host to danger (36).

The interaction between C3 and genomic DNA was confirmed also by ChIP sequencing and the major binding regions located into intergenic and intronic regions (Figure 6). Due to these results, we investigated if C3 interacts with histones, the structural proteins of DNA. DNA exists in two forms in the cell: euchromatin is prevalent in cells that are transcriptionally active while heterochromatin is most abundant in cells that are less or not active at all. The transition between eu- and heterochromatin is mediated mainly by changes in the histone proteins induced by interaction and/or competition with other proteins or by posttranslational modifications. Our results confirm a strong interaction between C3 and histone proteins, however—in contrast to DNA—the binding region was located mainly to the C3b part of the protein (Figures 7A–C) and C3a did not interact with the nucleic acid probably due to its highly cationic nature and the repulsion between the positive charges of both histones and C3a. On the contrary, C3a inhibited binding of the H1 linker histone to DNA (Figure 7D), revealing a potential mechanism how C3(a) binding to intronic regions might regulate gene transcription. Histone H1 binds to linker DNA of nucleosomes in a sequence independent manner, like C3, and plays a pivotal role in chromatin compaction and maintenance of the transcriptionally inactive, heterochromatin state. Many cationic peptides (like high mobility group antigens, receptor for advanced glycation end products) have been reported to compete with histone H1 for DNA binding and hence regulate the transition between hetero- to euchromatin, allowing or preventing gene transcription (37). In a similar way, the reduced association of histone H1 to DNA in the presence of C3a might have direct affect on DNA transcription and hence, regulate gene transcription. Although neither C3 nor its cleavage fragments induced direct chromatin remodeling in the FRET-based assay, it does not rule out that *ex vivo* or *in vivo*, C3 might interact with other nuclear factors to induce indirect epigenetic changes, which cannot be measured in cell-free systems. Hence, future experiments in a more complex system (i.e., KO mice and whole cell models) need to be carried out to reveal the exact functional consequence of C3—histone interaction in the nucleus.

Inherited C3 deficiency, a rare autosomal disease is associated with severe defect in dendritic cell maturation, improper memory B cell response and regulatory T cell development (6). The failure in initiating an effective adaptive immune response has been attributed to the absence of interaction between C3 activation products and their cognate receptors on the surface of adaptive immune cells. Our data evidencing the potential involvement of serum derived C3 in the regulation of gene transcription reveal a novel way by which serum C3 may influence adaptive immunity. Immunoprecipitation of C3 associated DNA material identified several potential targets which may be responsible for the defective immune response of C3 deficient patients (Figure 6). Among these, it is worth mentioning IL-7, a key cytokine involved in regulation of T cell responses (32), of which decreased level may cause also abnormal T-dependent B cell responses, two phenomena characteristic for C3-deficient patients. Similarly, altered expression of L-selectin, a key factor of lymphocyte adhesion and extravasation into secondary lymphoid organs (38) might explain the decreased immune response and opportunistic infections in the absence of C3.

Another interesting target of C3 might be complement receptor 1 (CR1). In contrast to freshly purified human B cells, malignantly transformed B cell lines lose CR1 due to unknown reason(s). Further, blood derived B cells rapidly decrease CR1 expression in cell culture (personal observations). Since the same phenomenon has been observed in the case of C3 uptake by immune cells, it is reasonable to assume that serum-derived and internalized C3 is involved in the regulation of CR1 expression on B cells. Similarly, a role for C3 in regulation of CR3 expression was observed via modulating the transcript level of CD11b integrins in mice (39). Although our study did not recruit C3 deficient patients, the altered distribution of distinct B cell subpopulations and expression of complement receptors (CR1/2 and CR3) in C3 KO mice (39) further support and point to the involvement of either C3-conveyed signals or the direct protein-DNA interaction in regulation of B cell responses and phenotype. Future analysis of the expression of C3 targeted genes and its correlation with disease pathology may directly contribute to the better understanding of the disease pathomechanism and hence, may directly facilitate the development of novel, complement-based therapies to restore immune homeostasis in the patients.

Taken together, our results reveal a so far unknown function of C3 whereby complement links innate and adaptive immunity and regulate basic processes of the cells. The identification of C3 targeted genes may help to understand disease pathology of C3 deficient patients and also pave the way for future studies to extend the homeostatic functions of the intracellular complement system. We are indeed only “just beginning to unravel this complex, intriguing, and important new system.”

## Data Availability

The results of ChIP sequencing can be found in Array Express database, E-MTAB-7506.

## Ethics Statement

This study was carried out in accordance with the recommendations of Ethics Committee in Lund with written informed consent from all subjects. All subjects gave written informed consent in accordance with the Declaration of Helsinki. The protocol was approved by the Ethics Committee in Lund (2017/582).

## Author Contributions

The experiments were designed by MK, and performed by MK, AN, PB, and FM. ChIP experiments were supervised by YD. Funding was obtained and project supervised by AB. The paper was written by MK and AB with input from all authors.

### Conflict of Interest Statement

The authors declare that the research was conducted in the absence of any commercial or financial relationships that could be construed as a potential conflict of interest.

## Abbreviations

A1AT: alpha-1 antitrypsin
BSA: bovine serum albumin
ChIP: chromatin immunoprecipitation
ChIPseq: ChIP sequencing
C3: complement C3
C3met: methylamine-treated C3
DGVB: D-glucose gelatin veronal buffer
GVB: gelatin veronal buffer
gDNA: genomic DNA
R: reducing (SDS-PAGE)
NHS: normal human serum
NR: non-reducing (SDS-PAGE).
