# Supplementary material for: Interaction of Serum-Derived and Internalized C3 With DNA in Human B Cells—A Potential Involvement in Regulation of Gene Transcription
Source: Front Immunol. 2019 Mar 19;10:493. doi: 10.3389/fimmu.2019.00493 (PMC6433827; doi:10.3389/fimmu.2019.00493)
Supplement: Supplementary file 1 [file Data_Sheet_1.docx]

Supplementary material

**Supplementary Figure 1.** Confirmation of C3 signal in human B cells.

Western blot results analyzing endogenous C3 expression of human B cells. Lysates prepared from the human B cell line, Raji were fractionated into cytoplasmic and membrane fractions and analyzed by Western blot with the goat polyclonal anti-C3 antibody from Quidel. As positive control, lysates prepared from mock, or C3-pcDNA3 transfected HEK293 cells and A549 cells (which express endogenously C3) were used. As negative controls, mock transfected HEK293 cell lysates or Crispr/Cas9 edited, C3 KO A549 cells were involved. As loading controls, the cytoplasmic marker B-tubulin and the membrane marker, Na/K ATPase were used. Results shown are representative of two independent experiments.

| 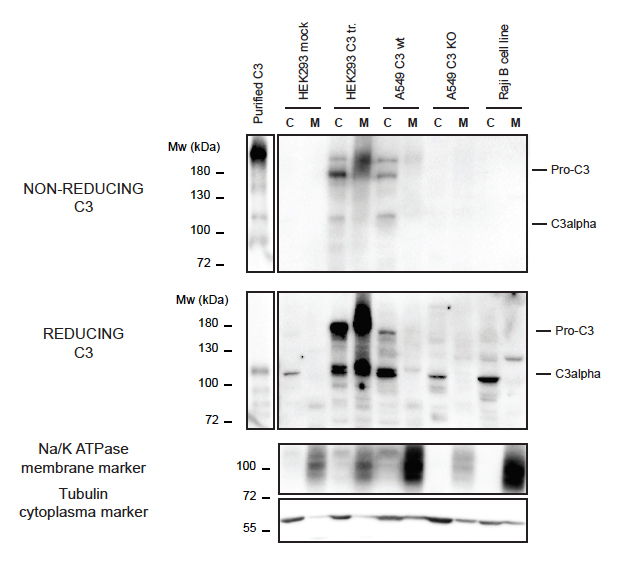 |
| --- |

**Supplementary Figure 2.** C3 uptake is common among immune cells.

Western blot results analyzing C3 uptake by the human Jurkat T cell line **(A)** and human monocytoid THP-1 cells **(B)**. Cells were treated with distinct sources of C3 (10 % NHS, 100 μg/ml C3, 100 μg/ml C3met) in DGVB++, Mg-EGTA or EDTA-GVB buffer for 1 h at 37 ^o^C. C3 uptake was investigated by Western blot using the goat polyclonal anti-C3 antibody from Quidel under non reducing conditions. Data shown are representative of two independent experiments.

| 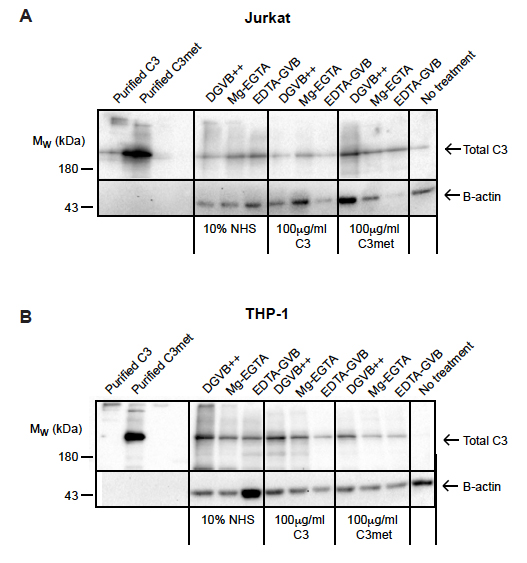 |
| --- |

**Supplementary Figure 3.** C3 loading to the nucleus is not restricted to B cells.

Western blot results showing presence of C3 in nuclear compartments of Jurkat cells. Cells were incubated either with NHS, C3 or C3met in EDTA-GVB buffer for 1 h at 37 ^o^C. After lysis, cytoplasmic and soluble nuclear fractions were separated and analyzed by Western blot with the goat polyclonal anti-C3 antibody under non reducing conditions. The purity of distinct cellular fractions was verified using antibodies against B-actin (cytoplasmic marker) and lamin B1 (nuclear marker). Data shown are results of one representative experiment out of two independent analyzis.

| 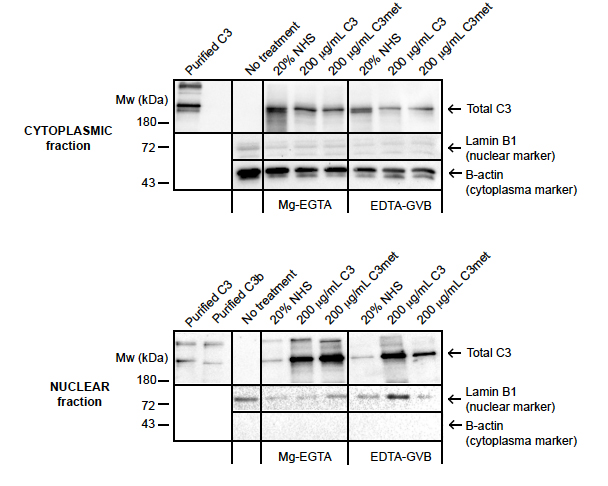 |
| --- |

**Original agarose gel electrophoresis and Western blot pictures**

| 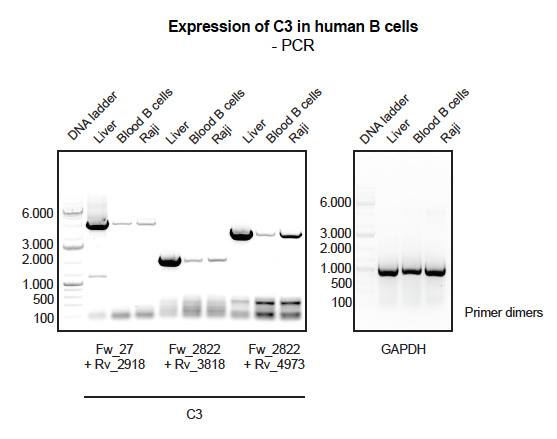 |
| --- |
| **Figure 1B.** Endogenous expression of C3 is very low in human B cells – PCR results. |

| 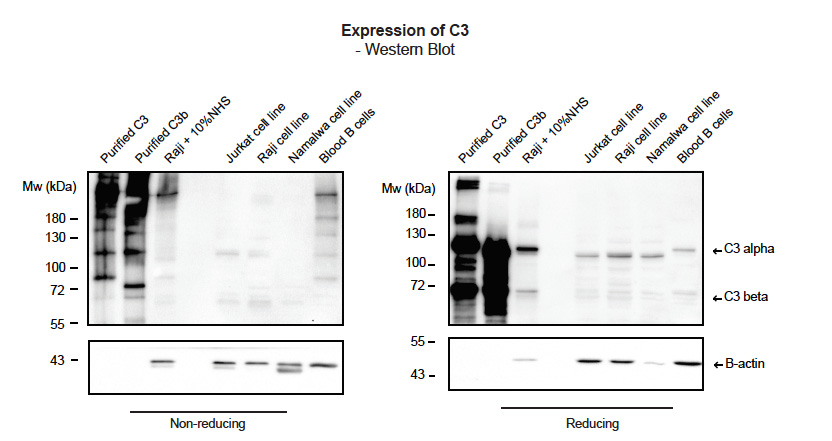 |
| --- |
| **Figure 1C.** Endogenous expression of C3 is very low in human B cells – Western blot results. Samples were run under non-reducing and reducing conditions. Membranes were cut around 50kDa – upper membrane developed with polyclonal goat anti-C3 antibody, lower developed with anti-B-actin antibody. |

| 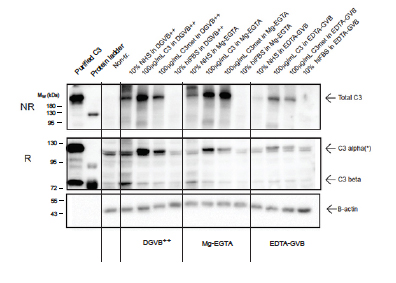 |
| --- |
| **Figure 2A** Internalization of C3 by human B cells. Samples were run under non-reducing (NR) and reducing ® conditions. Membranes, blotted after reducing SDS-PAGE, were cut around 60kDa – upper membrane developed with anti-C3 antibody, lower developed with anti-B-actin antibody. |

| 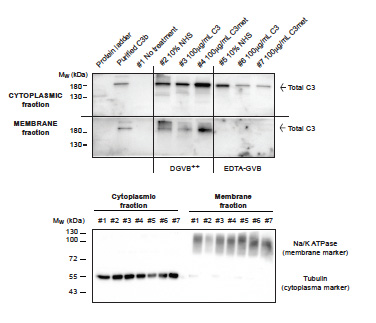 |
| --- |
| **Figure 2B** Internalization of C3 by human B cells – cytoplasmic/membrane fractionation of Raji cells. Samples were run under non-reducing condition (C3 Western blot) or under reducing condition to develop loading controls. C3 blots were cut around 100kDa, lower membrane – developed for loading controls – around 70kDa and developed either with anti-Na/K ATPase or anti-beta tubulin antibodies. |

| 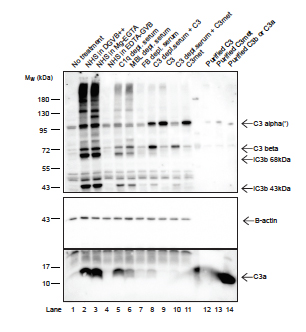 |
| --- |
| **Figure 3.** C3a in B cells is generated extracellularly by alternative pathway C3-convertases. Samples were run on reducing SDS-PAGE. In case of C3a detection, membranes were cut around 26kDa. Upper part developed with polyclonal rabbit anti-C3a, lower developed with anti-B-actin antibody. |

| 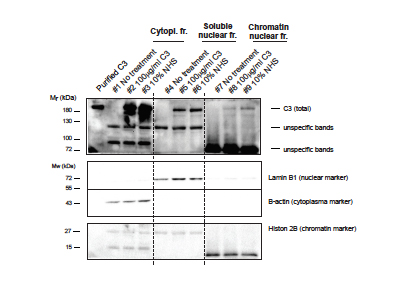 |
| --- |
| **Figure 4A.** Western blot results showing presence of C3 in nuclear compartments of Raji cells. Samples were run on non-reducing SDS-PAGE for C3 detection. To analyze the purity of cytoplasmic, membrane and chromatin-associated fractions, samples were run on reducing SDS-PAGE, membranes cut around 55kDa and 36kDa, and developed with anti-lamin B1 (upper), or anti-B-actin (middle) or histone 2B (lower) antibodies. |
| 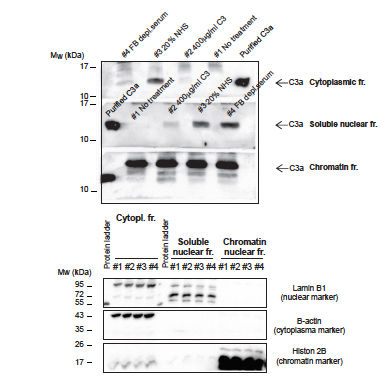 |
| **Figure 4B.** Western blot results showing presence of C3a in nuclear compartments of Raji cells. Samples were run on reducing SDS-PAGE, each fraction separately, membranes cut around 36kDa and lower part of the membranes were developed with the rabbit polyclonal anti-C3a antibody. To analyze the purity of cytoplasmic, membrane and chromatin-associated fractions, samples were run on reducing SDS-PAGE, membranes cut around 55kDa and 26kDa, and developed with anti-lamin B1 (upper), or anti-B-actin (middle) or histone 2B (lower) antibodies. |
